# Wearable woven supercapacitor fabrics with high energy density and load-bearing capability

**DOI:** 10.1038/s41598-017-14854-3

**Published:** 2017-10-30

**Authors:** Caiwei Shen, Yingxi Xie, Bingquan Zhu, Mohan Sanghadasa, Yong Tang, Liwei Lin

**Affiliations:** 1 University of California at Berkeley, Berkeley, CA 94720 USA; 2 School of Mechanical & Automotive Engineering, South China University of Technology, Guangzhou, Guangdong, 510641 China; 3 Department of Engineering Mechanics, Tsinghua University, Beijing, 100084 China; 4 Aviation and Missile Research, Development, and Engineering Center, US Army, Redstone Arsenal, AL 35898 USA

## Abstract

Flexible power sources with load bearing capability are attractive for modern wearable electronics. Here, free-standing supercapacitor fabrics that can store high electrical energy and sustain large mechanical loads are directly woven to be compatible with flexible systems. The prototype with reduced package weight/volume provides an impressive energy density of 2.58 mWh g^−1^ or 3.6 mWh cm^−3^, high tensile strength of over 1000 MPa, and bearable pressure of over 100 MPa. The nanoporous thread electrodes are prepared by the activation of commercial carbon fibers to have three-orders of magnitude increase in the specific surface area and 86% retention of the original strength. The novel device configuration woven by solid electrolyte-coated threads shows excellent flexibility and stability during repeated mechanical bending tests. A supercapacitor watchstrap is used to power a liquid crystal display as an example of load-bearing power sources with various form-factor designs for wearable electronics.

## Introduction

The rapid advancements of flexible and wearable electronics leads to a growing demand of reliable power supply. Lithium-ion batteries with high energy densities are the state-of-art solutions but they surfer from safety risks with poor compatibility with flexible systems^1,2^. Electrochemical supercapacitors (SCs), as the energy storage device bridging the gap between batteries and conventional capacitors, are promising candidates with safe operation, fast charging rates, long cycle life, and relatively simple configuration^3–6^. Unlike lithium-ion battery electrodes based on bulk reactions which cause structural failure after repeated charging and discharging operations, SCs electrodes that store energy through electrochemical double-layer capacitance are mechanically stable and allow for various flexible device designs with diverse choices of electrode materials^3–10^. Therefore, even with lower energy densities than rigid lithium-ion batteries, flexible SCs have the potential to enable new flexible and wearable electronics where flexibility and safety are of particular importance.

Recently, two types of flexible SCs with either two-dimensional (2D) or one-dimensional (1D) electrode configurations have been frequently reported. Flexible SCs using sandwiched or in-plane interdigital 2D thin-film electrodes always use fragile electrode materials supported by flexible substrates such as plastic membranes and textiles^4–6,9^. Though much progress has been made on the electrochemical performances of the electrode materials, the flexibility, mechanical strength, and stability of these devices rely heavily on additional mechanical support. For example, in practical applications, the substrates and packaging materials of these devices occupy a large portion of the total weight/volume and greatly diminish the overall energy density.

Several groups have reported flexible SCs based on coaxial, twisted, or parallel 1D electrodes to achieve high flexibility and functionality by integrating mechanical support, current collector, and capacitive electrode materials in a single fiber/yarn/thread^7,10,11^. However, issues such as high proportion of mechanical support still exist by coating functional nanomaterials onto heavy metal wires^12–14^ or non-conductive wearable fibers^15–20^. A promising approach is to develop dual-functional electrode materials that provide energy storage and mechanical support simultaneously. For example, researchers have demonstrated free-standing yarn electrodes by the spinning of carbon nanotubes, graphene oxide, conducting polymers, and so on^21–29^. Such yarns achieve high capacitances (up to ~300 F cm^−3^ per electrode) because of the absence of binder materials, current collector, and other supporting compositions^26^. However, their tensile strength, which decreases with the increase of porosity, is usually around 100 MPa, which is much lower than that of typical wearable fibers (~1000 MPa for cotton and polyester fibers^30^). As a result, they have to be assembled on a plastic substrate or woven into other textiles for practical applications^21,22,26^. Commercial carbon fibers (CF) with high mechanical strength (over 1000 MPa), light weight, and high conductivity are another promising structural material as 1D electrode for flexible SCs^31–35^. However, pristine CF has low surface area (<1 m^2^ g^−1^) and is usually used as an electrode to load other capacitive nanomaterials^31–34^, while surface-activated CF has a higher surface area but limited to less than 100 m^2^ g^−1^ ^35^.

Here we demonstrate directly-woven SC fabrics using nanoporous CF thread electrodes with both high surface area (340 m^2^ g^−1^) and high strength (1955 MPa) through a scalable fabrication process. The flexible SCs show a high energy density (2.58 mWh g^−1^ or 3.6 mWh cm^−3^, normalized by the whole weight/volume of the device) comparable to rigid commercial supercapacitors and can work under a high tensile strength of 1000 MPa or a high pressure of 100 MPa without noticeable performance degradations. The possible versatile SC configurations allow for a variety of form-factor designs of energy storage fabrics with excellent flexibility and mechanical stability.

## Results and Discussion

### Material and structure design

**Figure 1:**
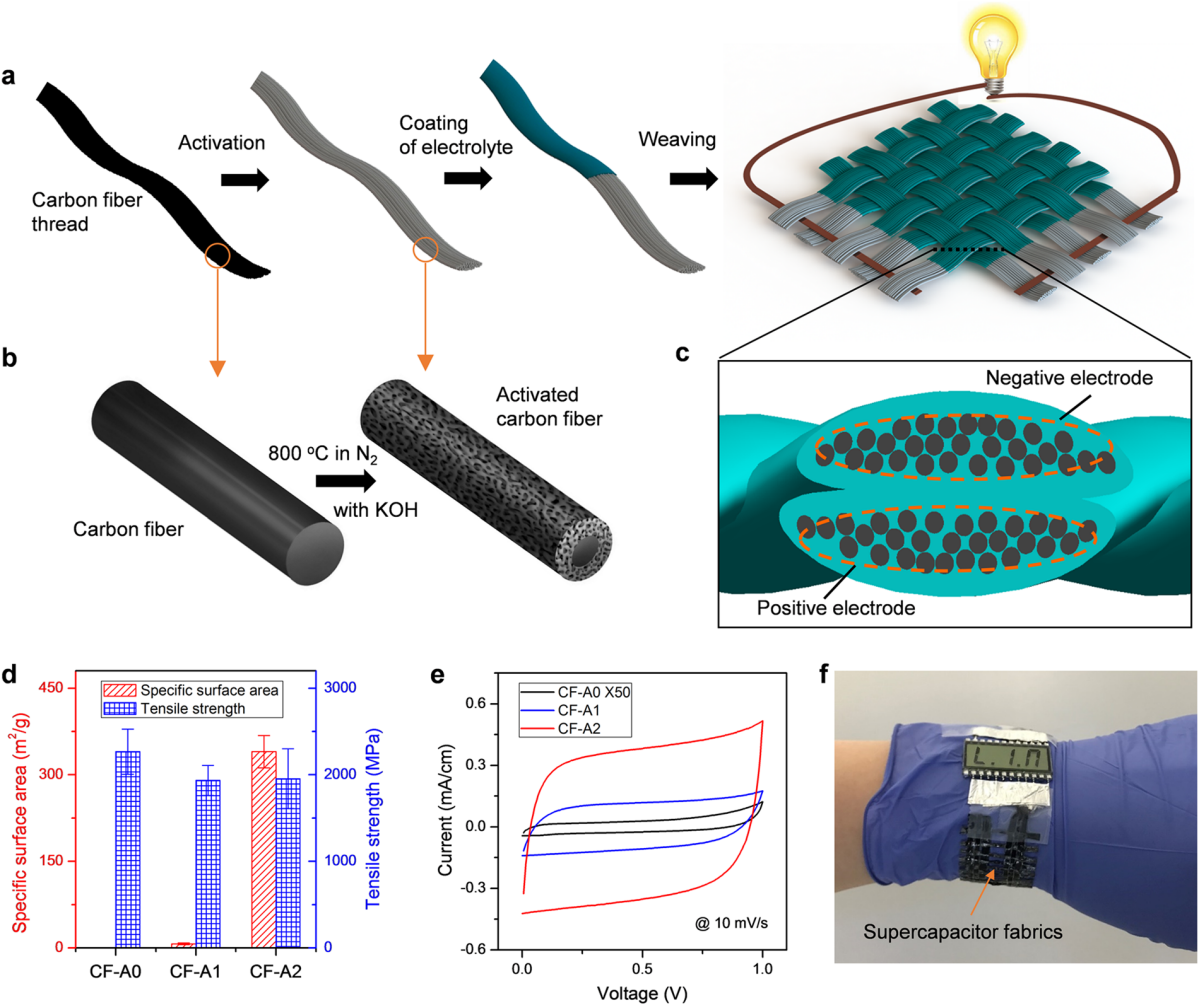
(**a**) Fabrication process for the load-bearing supercapacitor fabrics by using activated carbon fiber threads. (**b**) The activation process of a carbon fiber, after which the carbon fiber has a nanoporous surface and a solid core with high mechanical strength. (**c**) Cross-section schematics of the supercapacitor fabrics showing two crossing threads as positive and negative electrodes, respectively, with a solid electrolyte layer in between. (**d**) Measured specific surface area and tensile strength of carbon fiber threads with no-activation (CF-A0), after one-activation (CF-A1), and after two-activation (CF-A2) processes. The surface area increases dramatically after the two-activation process while the mechanical strength remains high. (**e**) The cyclic voltammetry curves showing the EDL capacitances of two-electrode cells using different carbon fibers tested in 1 M H_3_PO_4_ solution. The current is normalized by the length of the thread electrode dipped into the electrolyte solution. (**f**) A watchstrap made of load bearing supercapacitor fabrics powering a screen on a human wrist.

**Figure 2:**
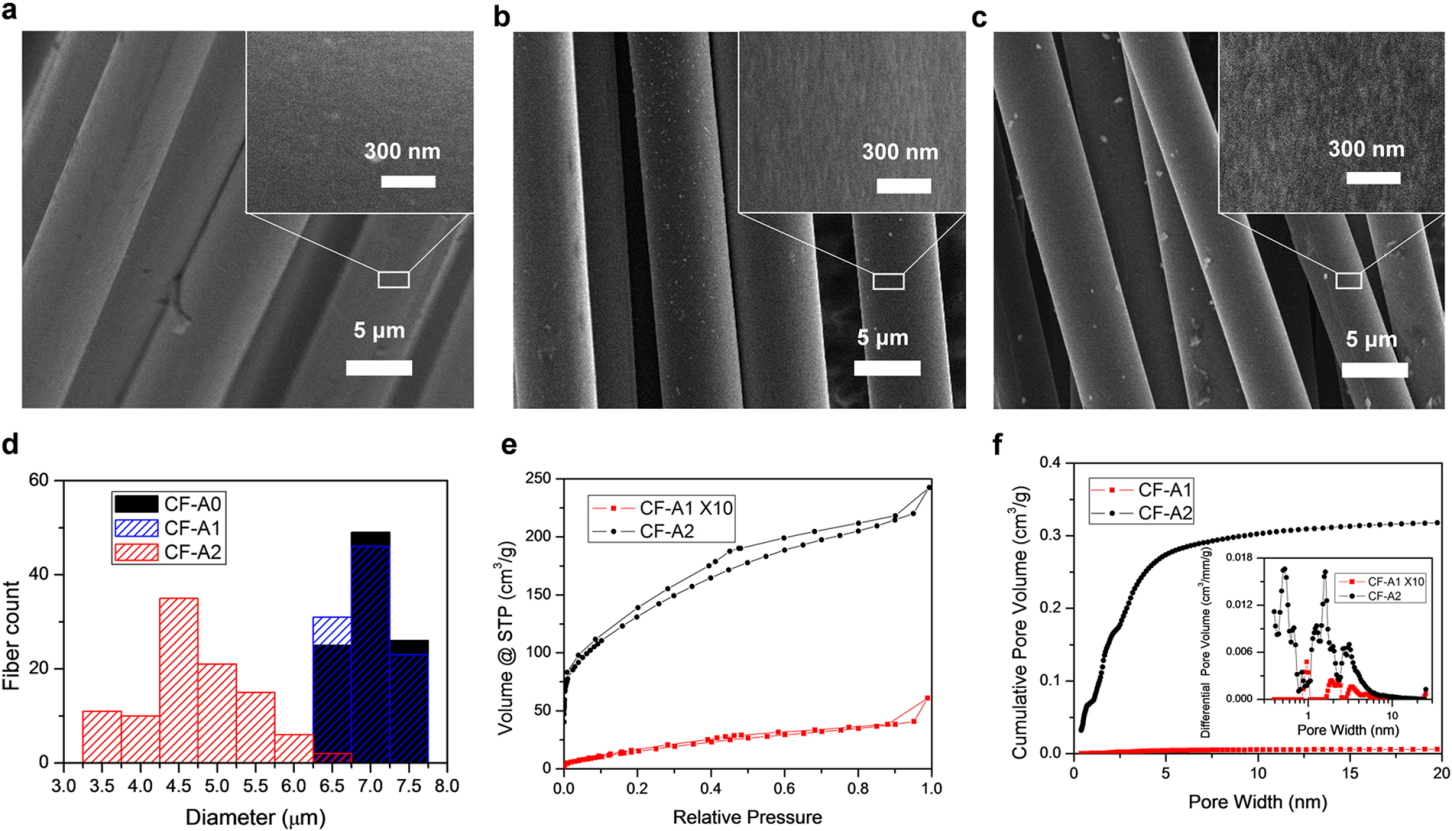
SEM images of carbon fiber threads: (**a**) with no activation (CF-A0), (**b**) after one activation (CF-A1), and (**c**) after two activations (CF-A2). (**d**) Diagram showing the diameter distribution of CF-A0, CF-A1, and CF-A2. (**e**) N_2_ adsorption/desorption isotherm of activated carbon fiber samples. (**f**) Cumulative pore volume and (inset) pore-size distribution of the samples calculated by using a slit/cylindrical nonlocal density functional theory (NLDFT) model.

The SC fabrics are woven by solid electrolyte-coated CF threads as illustrated in Fig. 1a. The nanoporous CF threads are prepared by a chemical activation process with potassium hydroxide (KOH, Fig. 1b, see Supplementary Information for details). The crossing threads in the fabrics, between which ions can shuttle through contacted electrolyte layers, are used as positive and negative electrodes, respectively (Fig. 1c) and the whole fabrics can be used as an electrochemical double layer (EDL) supercapacitor with symmetric electrodes. As-received commercial CFs (denoted as CF-A0) are in the form of continuous threads consisting of many graphite filaments with a diameter of ~7 μm (Supplementary Figure S1a, Fig. 2a). We have developed a two-step activation process to improve the specific surface area (SSA) of CF-A0. After the first activation process, the SSA has one-order of magnitude increase and after the second activation process (CF-A2), a three orders of magnitude increase in SSA has been observed. Furthermore, the mechanical strength is only reduced about 15% after the second activation process (Fig. 1d). As a result, CF-A2 shows high EDL capacitance when tested in a liquid electrolyte (Fig. 1e). Supercapacitor fabrics woven by such electrodes can be used as flexible power sources that store energy and bear loads at the same time, such as in a wearable powering watchstrap (Fig. 1f).

### Morphology and surface area of nanoporous carbon fibers

As-received commercial carbon fibers have smooth surface (Fig. 2a) and a measured SSA of 0.5 ± 0.3 m^2^ g^−1^. About 5 wt% of the carbon fiber is etched away after the one-time activation process (Supplementary Table S1), resulting in rough surface under the scanning electron microscope (SEM, Fig. 2b) and slight change in diameter distributions (Fig. 2d). After the two-time activation process, about 50 wt% of the carbon fiber is lost, which creates rougher surface (Fig. 2c) and thinner carbon fiber with wider diameter distributions (Fig. 2d). The N_2_ adsorption/desorption experiments are conducted to analyze the surface area and pore size distribution. The Brunauer–Emmett–Teller (BET) SSAs calculated from isotherms in Fig. 2e are 7.0 ± 1.5 m^2^ g^−1^ and 340 ± 28 m^2^ g^−1^ for CF-A1 and CF-A2, respectively, which are much larger than that of CF-A0. Cumulative pore volumes and pore-size distributions of CF-A1 and CF-A2 are compared in 2 f, showing that CF-A2 has much larger pore volume. The pore size of CF-A1 is mostly around 1 nm, and that of CF-A2 is smaller in average. This indicates that most of the surface area is contributed by micropores created by KOH activation^36^.

### Mechanical properties of electrodes

**Figure 3:**
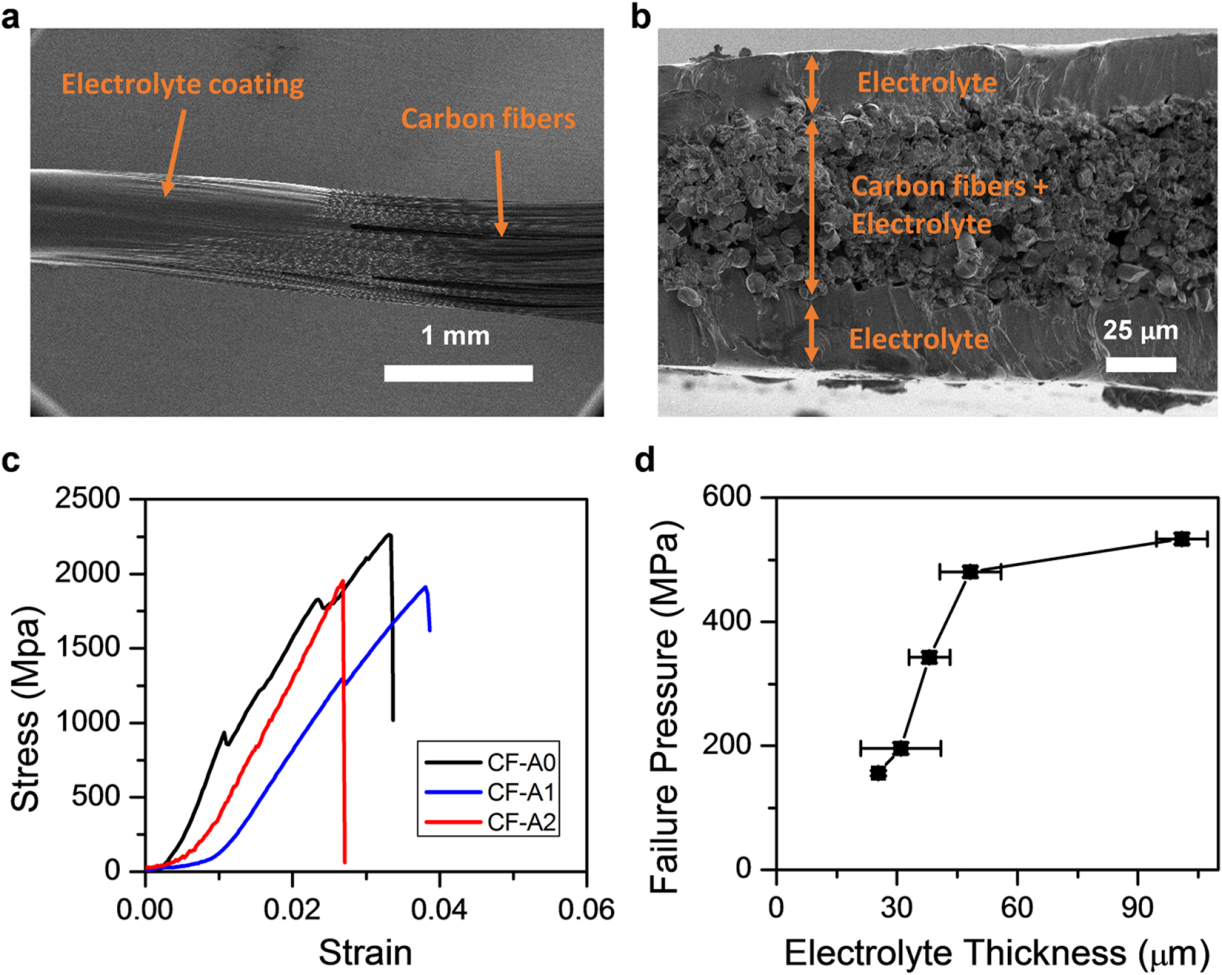
SEM images of (**a**) the top view and (**b**) the cross-section view of a carbon fiber thread containing 3000 fibers coated with solid electrolyte. (**c**) Tensile tests of threads using CF-A0, CF-A1, and CF-A2. (**d**) Failure pressure of the crossing point in supercapacitor fabrics as a function of electrolyte thickness. In each test, two identical threads with electrolyte coating are overlapped and compressed, and the pressure at which two threads are electrically shorted is recorded as the failure pressure.

After the activation processes, a polymer-based electrolyte solution is coated on the carbon fibers by impregnation and gradually dried to form a solid electrolyte layer that binds carbon fibers together (Fig. 3a). A drum is rolled over the thread during the drying process to adjust the thickness of the electrode and the coating process can be repeated to coat a thicker electrolyte layer. Figure 3b shows the cross-section SEM image of a sample with ~70 μm-thick electrode (carbon fibers + electrolyte) and ~25 μm-thick electrolyte after twice coating of electrolyte. In the woven supercapacitor fabrics, the uniform electrolyte layer can prevent the crossing of electrodes from shorting such that no separator is needed. The typical stress-strain curves of threads using CF-A0, CF-A1, and CF-A2 are plotted in Fig. 3c. Although the tensile strength of pristine CF is 3530 MPa from the data sheet of the product^37^, the measured average strength of CF-A0 is ~2266 MPa because not all carbon fibers in a thread are stretched to the same extent during the test (see test sample photo in Supplementary Figure S1b). Variations of up to ±18% of the values are measured in different threads, as summarized in Fig. 1d. For the same reason, each thread shows a slight difference in the stress-strain behavior and the failure strain. Decrease of tensile strength is found in the activated fibers as compared with original ones. The average tensile strengths of CF-A1 and CF-A2 are 1936 ± 173 MPa and 1955 ± 345 MPa, respectively, which are 85% and 86% of CF-A0, all much higher than those of wearable fibers^30^. In spite of tensile stress, the supercapacitor may fail due to compressive force that breaks the electrolyte layer and shorts the two electrodes. Considering this, the polymer with relatively high molecule weight is chosen to provide high compressive strength and a simple setup is designed for experimental data (Supplementary Figure S1c,d). Figure 3d plots the failure pressure of the electrolyte layer as a function of thickness. High failure pressures from 156 to 534 MPa are measured for electrolyte thicknesses from 25 to 100 μm, respectively. The increase of electrolyte thickness clearly reduces the probability of short between the crossing electrodes caused by compressive force. However, thick electrolyte layer can increase the ionic resistance and decrease the power performance and the balance between high power density and high resistance for compressive strength can be designed based on the specific application.

### Electrochemical performance of woven supercapacitor fabrics

**Figure 4:**
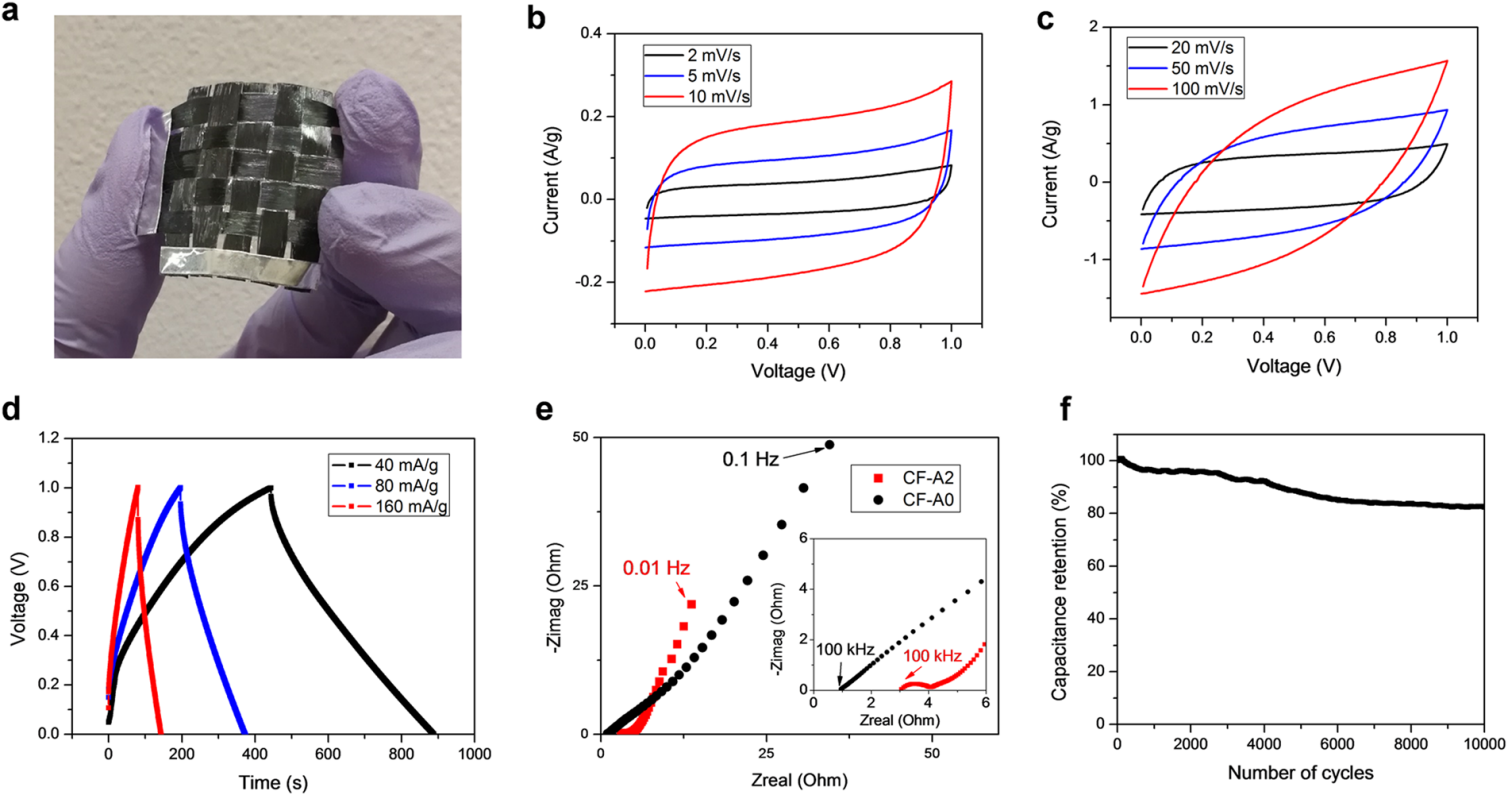
(**a**) A photo of a load bearing supercapacitor fabric with the size of 2.5 cm × 2.5 cm. Cyclic voltammetry (CV) curves of the supercapacitor fabric at (**b**) lower scan rates and (**c**) higher scan rates with current normalized to the total weight of all carbon fiber threads (~25 mg). (**d**) Galvanostatic charge–discharge curves of the device under various current densities (normalized to the total weight of all carbon fiber threads). (**e**) Nyquist plots of the impedances of the woven devices using CF-A0 and CF-A2 with the frequency range from 0.01 to 100k Hz. (**f**) Capacitance retention of the device using CF-A2 during 10,000 charge/discharge cycling tests.

The solid electrolyte-coated carbon fiber threads can be woven into supercapacitor fabrics using a loom or by hand (Supplementary Figure S2). Samples of various sizes up to ~100 cm^2^ have been prepared and tested, showing capacitance increasing with the size (Supplementary Figure S3). A representative handwoven supercapacitor fabric sample using CF-A2 is shown in Fig. 4a. Cyclic voltammetry (CV) curves of the sample in Fig. 4b and c display a quasi-rectangular shape at lower scan rates from 2 mV s^−1^ to 20 mV s^−1^, with current increasing almost linearly with scan rate, which indicates excellent EDL capacitance behavior. Deviation from a rectangular shape happens at higher scan rates of 50 mV s^−1^ and 100 mV s^−1^ due to low ionic conductivity of the solid electrolyte that limits the speed of charge/discharge. Galvanostatic charge–discharge (GCD) curves in Fig. 4d exhibit a nearly triangular shape for various current densities. Slight deviation of charge and discharge curves from straight lines is also caused by relatively slow ion transport. Equivalent series resistance estimated from the IR drop is ~0.81 Ω g, which is mostly contributed from the ionic resistance because the electrical resistance of carbon fiber threads is measured to be less than 0.02 Ω g. Another evidence is that the ionic conductivity of the solid electrolyte is measured to be ~0.081 S m^−1^, much smaller than the electronic conductivity of the CF electrode (~5.8 × 10^4^ S m^−1^). More information on the impedance of the device is shown in the Nyquist plot in Fig. 4e. For comparison, the impedance of a fabric device woven by CF-A0 is also plotted in the same graph. Capacitive behavior is dominating at low frequencies for both devices as indicated by the rapid change of the imaginary part. The curve is more inclined at middle frequency range because of the diffusion impedance (Warburg impedance) associated with slow ion diffusion in the solid electrolyte. A semicircle at higher frequency range, which is attributed to the transport processes of both electrons and ions in nanoporous electrodes^38^, is presented for the device using CF-A2, but is not seen for the one using CF-A0 with no nanoporous structures.

Specific capacitances of 18.6 F g^−1^ and 17.8 F g^−1^, corresponding to energy densities of 2.58 mWh g^−1^ and 2.47 mWh g^−1^, are calculated from the CV curve at 2 mV s^−1^ and the GCD curve at 40 mA g^−1^, respectively (normalized to the total weight of the fabric). The energy density as a function of power density is also calculated and plotted in Supplementary Figure S4. Such energy densities are comparable to those of rigid commercial supercapacitors which are usually 1 to 10 mWh g^−1^ ^39,40^. If the total area of the fabric (2.5 cm × 2.5 cm) and the density of the electrolyte-coated thread electrode (~1.4 g cm^−3^) are calculated, the SC fabric provides areal capacitance of ~300 mF cm^−2^ and energy density of ~42 μWh cm^−2^, or volumetric capacitance of ~26 F cm^−3^ and energy density of ~3.6 mWh cm^−3^ (all the metrics are normalized to the total volume/areal of the fabric).

Electrochemical stability of the sample device is tested and over 80% of the capacitance is retained after 10000 cycling tests at a scan rate of 20 mV s^−1^ (Fig. 4f). The test is done without any encapsulation, and the drop of capacitance can be resulted from the loss of water in the electrolyte^41^. For practical applications, simple treatment can be used to make such SC fabrics waterproof without losing performance. For example, a very thin and conformal coating of parylene (~800 nm) through chemical vapor deposition can serve as a protection layer, and the sample device retains its energy storage property after dipped in water for 1 hour (Supplementary Figure S5).

### Mechanical tests and flexibility of supercapacitor fabrics

**Figure 5:**
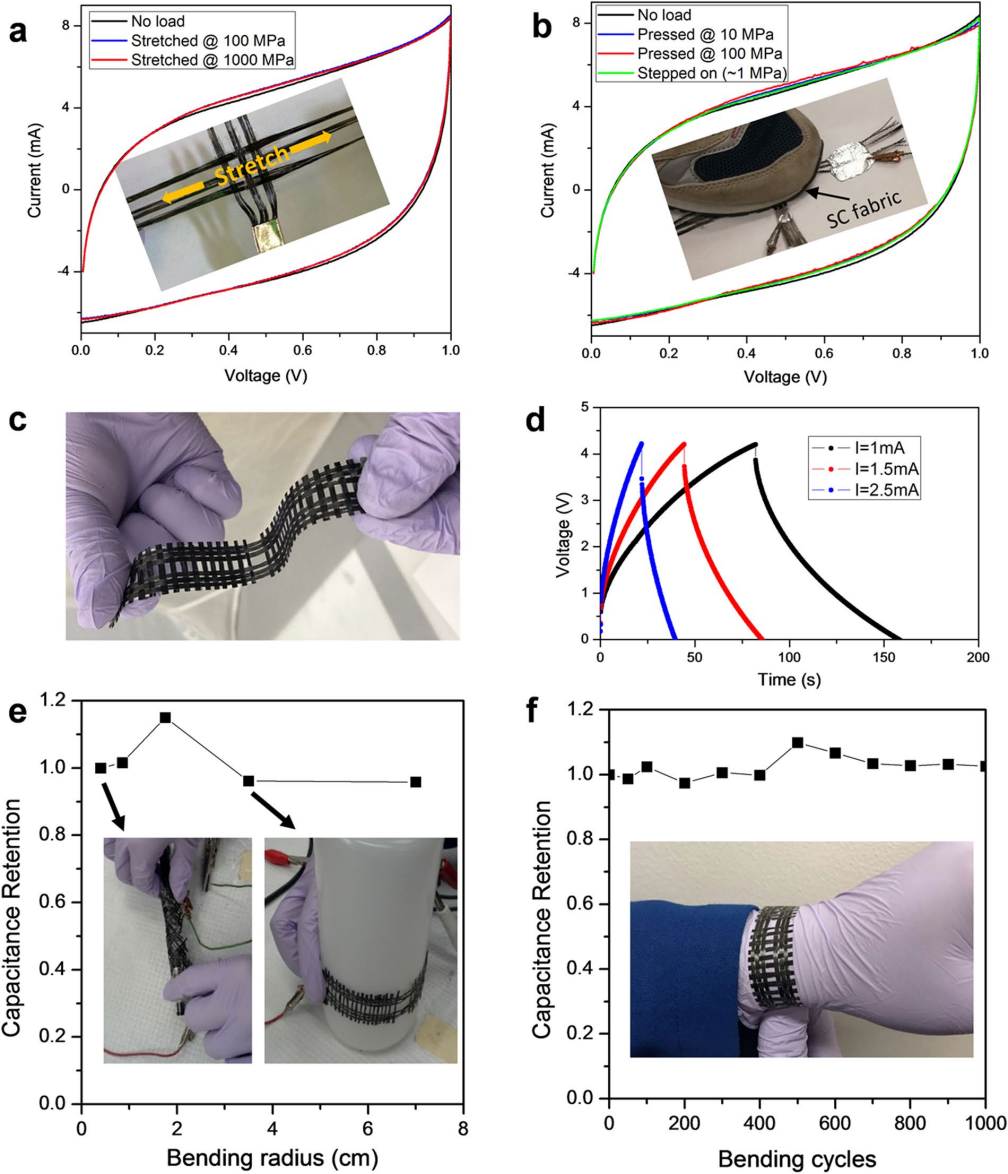
(**a**) CV curves of a supercapacitor fabric (as shown in the inset photo) when stretched at different stresses at the scan rate of 20 mV s^−1^. (**b**) CV curves of the sample when pressed with different pressures at the scan rate of 20 mV s^−1^. The inset photo shows how it is tested when the sample is stepped on. (**c**) A photo of a load bearing supercapacitor strap with the 4-cell connect-in-series design. (**d**) Galvanostatic charge–discharge tests of the strap working at 4 V. (**e**) Capacitance retention of the strap under different bending radii. (**f**) Capacitance retention of the strap during 1000 bending cycles around a human wrist.

Wearable devices are constantly under tensile and compressive forces in practical uses such that most flexible energy storage devices require support and protection with substrates and packages, which occupy a large portion of the overall volume/weight. Furthermore, their electrochemical performances under high tensile and compressive loads, however, are seldom reported. In this work, the electrodes and electrolyte alone can serve as the load-bearing substrate without extra package. Figure 5a shows the CV curves of a SC fabric when it is stretched at a high tensile stress of 1000 MPa with almost no change in electrochemical performance. The tensile stress of 1000 MPa, however, will break most of the wearable fibers^30^ and functional fibers with energy storage properties made of CNT, Graphene, etc.^22,26,27^. Furthermore, the tensile strength of the woven supercapacitor fabrics qualifies the most stringent requirements in the automobile industry as the “GigaPascal steel” for Advanced High-Strength Steels (AHSS) with tensile strength of at least 1000 MPa^42^. The SC fabric also shows unchanged performance under a high pressure of 100 MPa (Fig. 5b). For example, a man of ~70 kg standing on an area of ~7 cm^2^ would generate a pressure of ~1 MPa and a failure pressure over 100 MPa would have a high built-in safety factor of 100.

In addition to high mechanical strength and stability, a variety of form-factor designs can be constructed. For example, a SC strap with high flexibility is woven as shown in Fig. 5c. A simple design is made so that one strap consists of four SC cells connected in series to work at a higher voltage of 4 V (Fig. 5d, Supplementary Figure S6). The electrochemical performance is tested when the strap was bent at different states, including coiled on a pen or a plastic bottle without showing significant changes under various bending radii (Fig. 5e). A possible application of such SC straps is a power watchstrap (Figs 1 f and 5f). The capacitance retention of the SC strap is tested for 1000 put-on/take-off cycles on a human wrist, corresponding to a bending radius of ~2.5 cm without losing its performances in Fig. 5f.

## Conclusions

In conclusion, direct-weave flexible SC fabrics with both high energy density and load-bearing properties are demonstrated by using multifunctional nanoporous CF threads that work as mechanical support, current collector, and capacitive material simultaneously. The thread electrodes are prepared by KOH activation of commercial carbon fibers with three orders of magnitude increase in the specific surface area and 86% retention of tensile strength. Combined with the directly woven electrode configurations, the SC fabrics feature scalable fabrication, arbitrary form-factor designs, excellent flexibility, and good mechanical stability as flexible power sources for wearable electronics.

## Methods

### Activation of carbon fiber threads

In a typical process, commercial CF threads, each containing 3000 filaments (TORAYCA^®^ T300), were cut and washed successively with acetone, isopropyl alcohol, and deionized water, and denoted as CF-A0 after drying. To prepare CF-A1, two steps including one KOH activation process and one reheating process were followed. For the KOH activation process, CF-A0 was dipped in 20 M potassium hydroxide (KOH) solution, and then heated in a tube furnace at 800 °C in N_2_ atmosphere for 30 min. The activated threads were washed with water until the pH value reached 7. For the reheating process, the washed threads were heated again at 800 °C in N_2_ atmosphere for 30 min. To prepare CF-A2, three steps including two KOH activation processes and one reheating process was used as described above.

### Coating of solid electrolyte and weaving of supercapacitor fabrics

The polymer-based electrolyte solution was prepared by dissolving 1 g of polyvinyl alcohol with high molecule weight (PVA, Mw = 146,000–186,000, Sigma-Aldrich^®^) in 12 g of deionized water at 80 °C, with 1 g of phosphoric acid (H_3_PO_4_) added and stirred until the solution became clear. The electrolyte was coated onto CF threads by impregnation, in which certain parts of the threads were drenched with electrolyte solution and then rolled over by a drum while drying. The coating process was repeated more than twice to ensure complete coverage. The electrolyte-coated threads were woven into fabrics as illustrated in Fig. 1a. At least one end of the threads is not coated with electrolyte so that electrical connection can be made.

### Characterizations

The SEM images were taken using a Quanta Scanning Electron Microscope. To perform tensile tests, the ends of the CF threads were bonded with fast-drying epoxy and clamped tightly by metal plates. The samples were then tested with a tensile testing machine (Instron^®^ Model 4483). The tensile stress was calculated by dividing the applied force by the cross-section area of carbon fiber threads. The cross-section area is estimated by the number of carbon fibers (3000 in this work) times the average cross-section area of a single carbon fiber filament ($\pi {r}^{2}$, where *r* is the root mean square of radii measured using SEM) with errors considered. The compressive tests were performed by using a hydraulic press (Carver^®^ Model 3912). Specific surface areas and pore size distributions were tested and calculated using QuadraSorb. The cyclic voltammetry, electrochemical impedance spectroscopy, and galvanostatic charge–discharge measurements were performed using a Gamry Ref 600 electrochemical station. Two-electrode setup was always used in this work, as three-electrode configuration often overestimates the capacitance of electrodes working in a full device^36^. A typical cyclic voltammetry curve of the CF-A2 sample was shown in Supplementary Figure S7 as an example.

## Electronic supplementary material

Supplementary Information
