# Supplementary material for: Wearable woven supercapacitor fabrics with high energy density and load-bearing capability
Source: Sci Rep. 2017 Oct 30;7:14324. doi: 10.1038/s41598-017-14854-3 (PMC5662724; doi:10.1038/s41598-017-14854-3)
Supplement: Supplementary file 1 — Supplementary Information [file 41598_2017_14854_MOESM1_ESM.pdf]

Supplementary Information

Wearable woven supercapacitor fabrics with high energy density and load-bearing capability

Caiwei Shen[1], Yingxi Xie[1,2], Bingquan Zhu[1,3], Mohan Sanghadasa[4], Yong Tang[2] and Liwei Lin[1,*]

[1]University of California at Berkeley, Berkeley, CA 94720, USA

[2]School of Mechanical & Automotive Engineering, South China University of Technology, Guangzhou, Guangdong 510641, China

[3]Department of Engineering Mechanics, Tsinghua University, Beijing 100084, China

[4]Aviation and Missile Research, Development, and Engineering Center, US Army, Redstone Arsenal, AL 35898, USA

* Email: lwlin@berkeley.edu

Table S1. Properties of different CF threads

Sample	Weight per length (mg/cm)	Weight loss*	Surface area (m^2/g)	Capacitance** (F/g)
CF-A0	2	0	0.5	0.048
CF-A1	~1.9	~5%	7.0	25.3
CF-A2	~1.0	~50%	340	78.6

* Weight loss compared to as-received commercial carbon fiber threads

** Tested in a symmetric two-electrode setup in 1M H_3PO_4 solution using cyclic voltammetry at 2mV/s, the values are normalized by the weight of each electrode dipped in the electrolyte

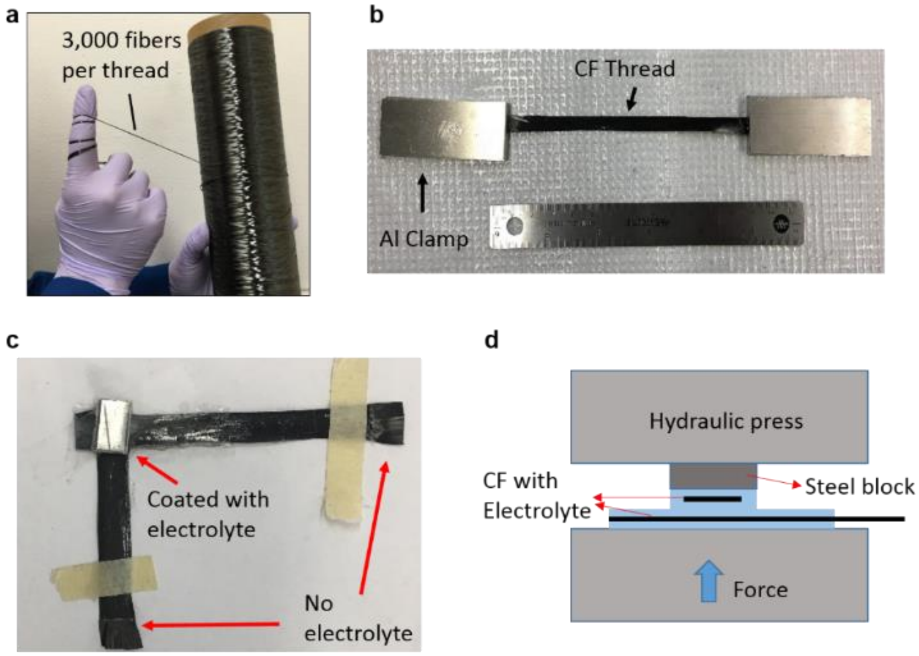

Figure S1: (a) A photo of as-received carbon fiber thread with 3000 filaments (fibers). (b) A photo showing the sample used for tensile strength tests. (c) A photo of the top view of a sample used for compressive strength test. (d) Schematic of the cross-section of the compressive test setup, the sample is pressed until the electrolyte layer breaks and the two CF threads are in contact with each other to cause the electrical short circuit.

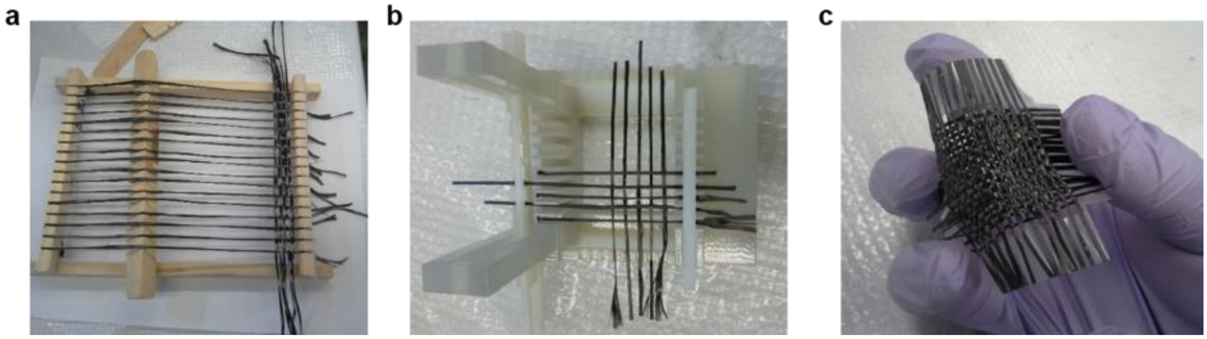

Figure S2: Photos showing the weaving of SC fabrics by using (a) a toy loom, (b) a small 3D-printed loom, and (c) a sample woven by hand.

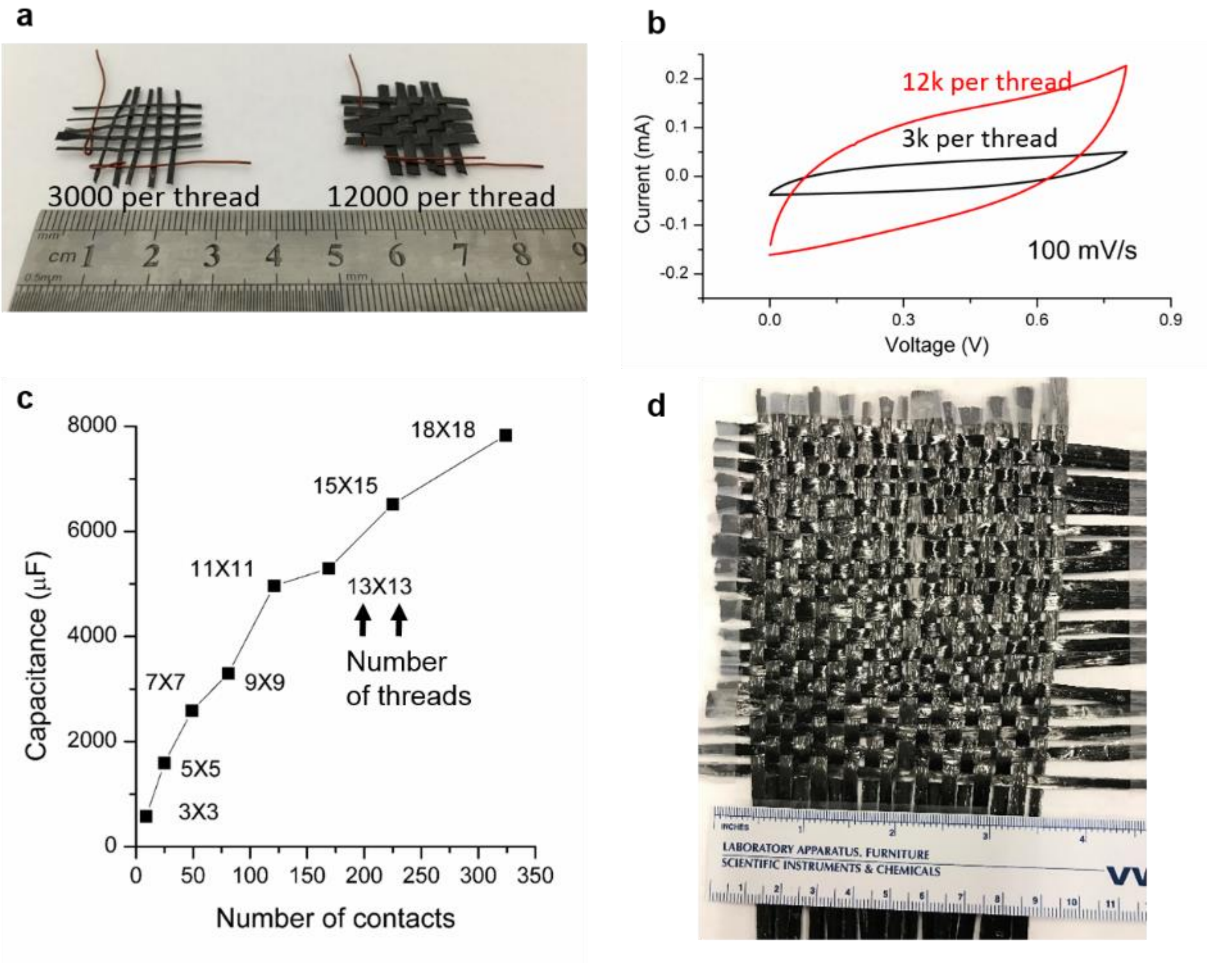

Figure S3: (a) A photo of two samples woven by threads of 3000 (3k) filaments and 12000 (12k) filaments, respectively. (b) CV curves of the two samples showing that the sample with 4 times of filaments per thread has 4 times of higher capacitance, proving that the capacitance is proportional to the total surface area of the electrodes. (c) The capacitance of SC fabrics as a function of number of contacts (crossing points). All the samples are woven by CF-A0. (d) A photo of a sample woven by 18 × 18 threads with an area of ~100 cm^2.

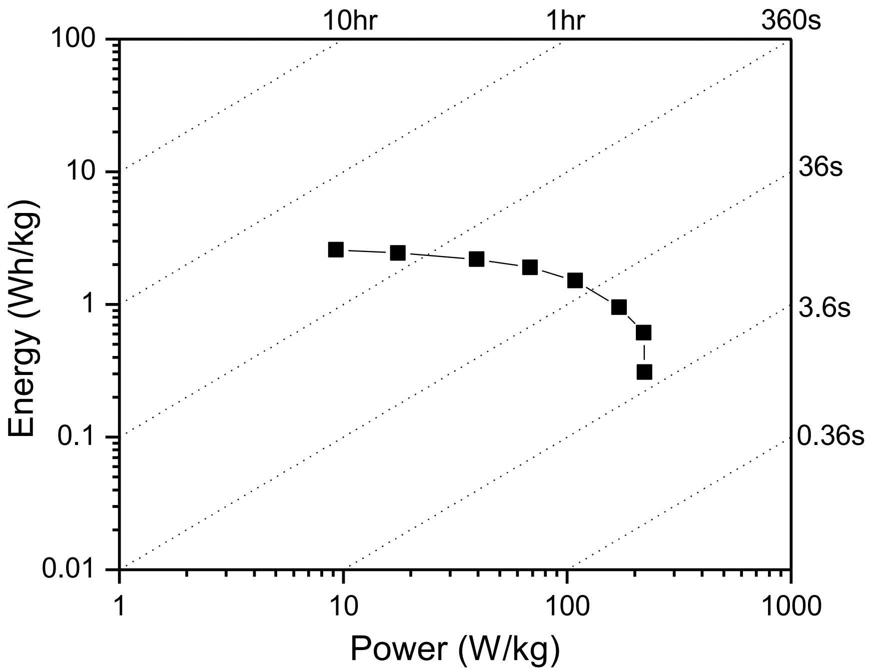

Figure S4: Ragone plot of the woven supercapacitor fabric shown in Figure 4a.

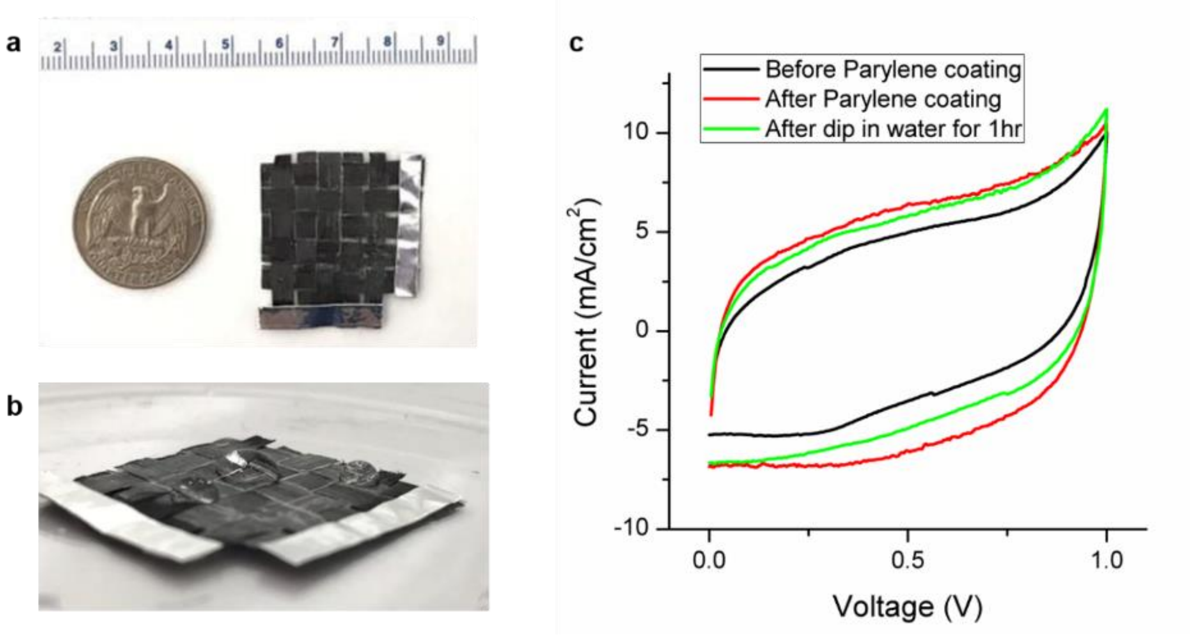

Figure S5: (a), (b): Photos of a waterproof SC fabric coated with an 800 nm-thick Parylene layer. (c) CV curves of the sample before and after the Parylene coating process, and after dipping into water for 1 hour, at a scan rate of 20 mV/s.

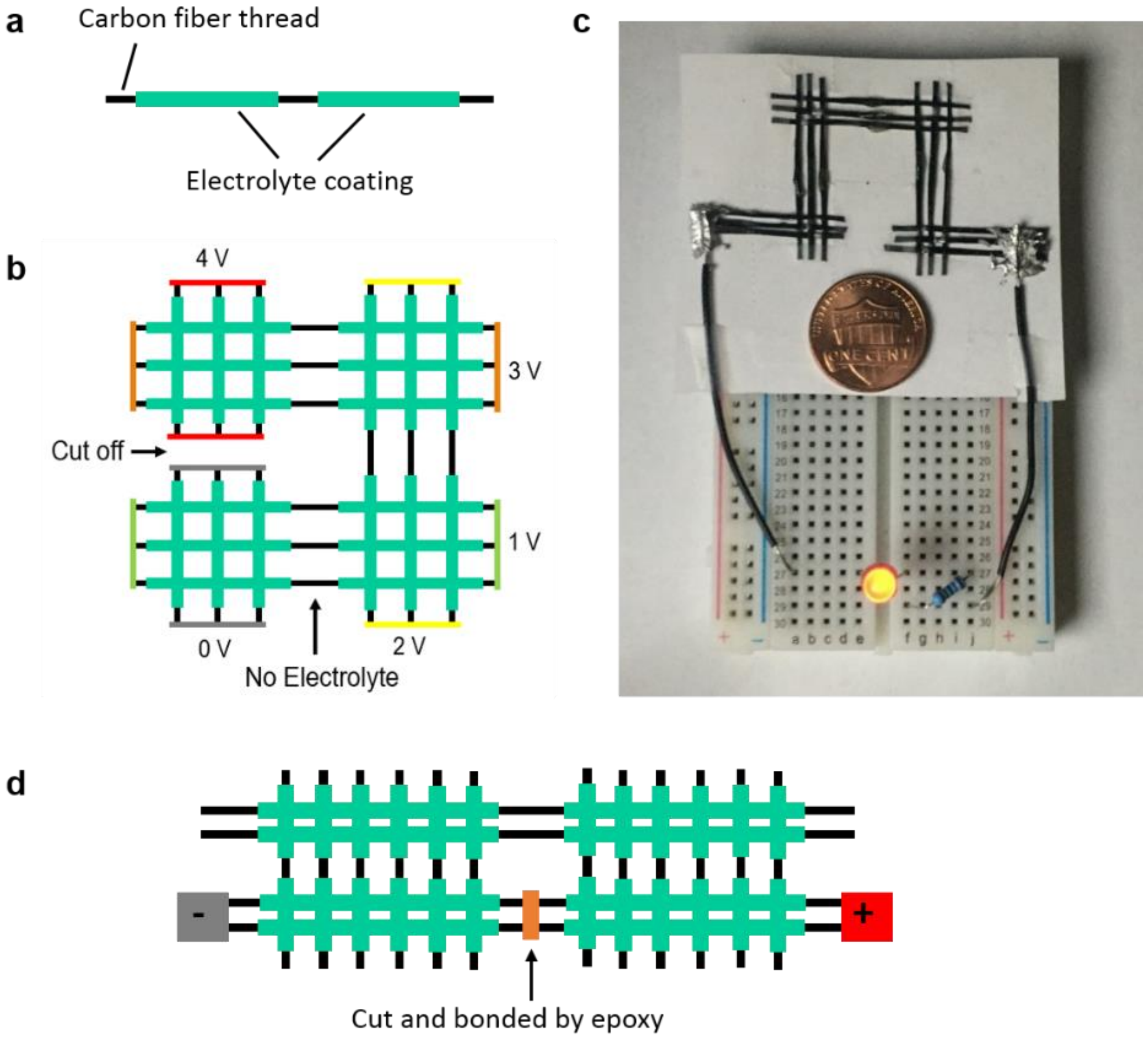

Figure S6: Schematics of (a) a carbon fiber thread that is partially coated with electrolyte; (b) a 4-cell-connect-in-series fabric woven by CF threads. (c) A photo showing an LED powered by a 4-cell-connect-in-series fabric. (d) Schematic showing the design of a SC strap with 4 cells connected in series.

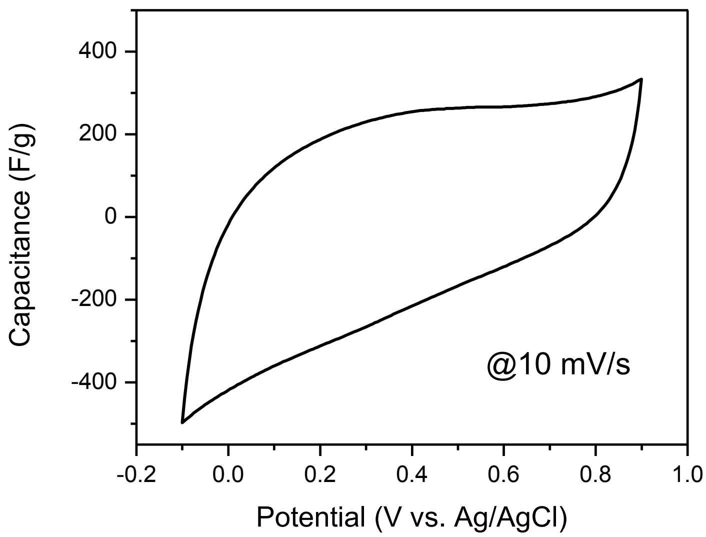

Figure S7: The cyclic voltammetry curve of CF-A2 electrode tested in a three-electrode setup with platinum wire as the counter electrode, Ag/AgCl electrode as the reference, and 1M H_3PO_4 solution as the electrolyte. The average capacitance of the CF-A2 electrode is calculated to be 198 F/g at 10 mV/s, much higher than the value estimated in two-electrode setup.

Equations Used for Performance Calculation:

For Cyclic Voltametry:

Scan rate: $r = \frac{dV}{dt}$

Capacitance: $C = \frac{\int Idt}{\Delta V} = \frac{\int IdV}{\Delta V \times r}$

Energy: $E=1/2\ CV^2$

For Galvanostatic charge/discharge:

Constant current: I

Capacitance: $C = \frac{I \Delta t}{\Delta V}$

Energy: $E=1/2\ CV^2$

$$Gravimetric\ C(or\ E, P) = \frac{C(or\ E, P)}{Total\ mass\ of\ the\ device}$$
